# Citizenship Matters: Non-Citizen COVID-19 Mortality Disparities in New York and Los Angeles

**DOI:** 10.3390/ijerph19095066

**Published:** 2022-04-21

**Authors:** Jason A. Douglas, Georgiana Bostean, Angel Miles Nash, Emmanuel B. John, Lawrence M. Brown, Andrew M. Subica

**Affiliations:** 1 Department of Health Sciences, Crean College of Health and Behavioral Sciences, Chapman University, Orange, CA 92866, USA; 2 Sociology and Environmental Science & Policy Programs, Schmid & Wilkinson Colleges, Chapman University, Orange, CA 92866, USA; gbostean@chapman.edu; 3 Donna Ford Attallah College of Educational Studies, Chapman University, Orange, CA 92866, USA; milesnash@chapman.edu; 4 Department of Physical Therapy, Crean College of Health and Behavioral Sciences, Chapman University, Irvine, CA 92618, USA; john@chapman.edu; 5 School of Pharmacy, Chapman University, Irvine, CA 92618, USA; lbbrown@chapman.edu; 6 Department of Social Medicine, Population & Public Health, Riverside School of Medicine, University of California, Riverside, CA 92521, USA; andrew.subica@medsch.ucr.edu

**Keywords:** COVID-19, non-citizens, Black and Latinx, health disparities, systemic racism, social determinants of health

## Abstract

U.S. non-citizen residents are burdened by inequitable access to socioeconomic resources, potentially placing them at heightened risk of COVID-19-related disparities. However, COVID-19 impacts on non-citizens are not well understood. Accordingly, the current study investigated COVID-19 mortality disparities within New York (NYC) and Los Angeles (LAC) to test our hypothesis that areas with large proportions of non-citizens will have disproportionately high COVID-19 mortality rates. We examined ecological associations between March 2020–January 2021 COVID-19 mortality rates (per 100,000 residents) and percent non-citizens (using ZIP Code Tabulation Areas (ZCTA) for NYC and City/Community units of analysis for LAC) while controlling for sociodemographic factors. Multiple linear regression analyses revealed significant positive associations between the percentage of non-citizen residents and COVID-19 mortality rates in NYC (95% CI 0.309, 5.181) and LAC (95% CI 0.498, 8.720). Despite NYC and LAC policies intended to provide sanctuary and improve healthcare access for non-citizen residents, communities with larger proportions of non-citizens appear to endure higher COVID-19 mortality rates. The challenges that non-citizens endure—e.g., inequitable access to public benefits—may discourage help-seeking behaviors. Thus, improved health surveillance, public health messaging, and sanctuary policies will be essential for reducing COVID-19 mortality disparities in communities with large shares of non-citizens.

## 1. Introduction

The COVID-19 pandemic in the United States has highlighted persistent health disparities in underserved communities, with Black and Latinx communities bearing a disproportionate burden of coronavirus-related morbidity and mortality [1,2]. While the rapid spread of this disease depicts a startling public health dilemma, the history of pandemic-related racial and ethnic health disparities in underserved U.S. communities indicates a pressing need to identify and prioritize at-risk populations for healthcare and COVID-19 treatment resource delivery to mitigate the disparate impacts of current and future viral pandemics [3,4]. For example, concerning racial and ethnic health disparities have existed for influenza since the 1918 influenza pandemic, when the U.S. mortality rate was 35% higher for persons of color compared to White persons. Between 1929 and 1939, the influenza death rate per 100,000 persons of the population was 71.3 for persons of color compared to 30.3 for White persons while in 1950, the age-adjusted influenza and pneumonia mortality rate per 100,000 persons of the population was 76.7 for African Americans vs. 44.8 for White individuals [3,5].

Improvements in public health surveillance have subsequently revealed that foreign-born U.S. residents may be at heightened risk for viral exposure during global pandemics. For example, “Spanish language-dominant” Latinx residents were at heightened risk of exposure during the H1N1 pandemic [6]. More recently, research examining COVID-19-related deaths in California and Minnesota indicated that foreign-born Latinx U.S. residents may be at heightened risk for COVID-19-related mortality [7,8].

These recent insights question the immigrant health paradox, which argues that U.S. immigrants generally experience better health outcomes than native citizens [9]. However, a growing body of evidence suggests that health-protective factors associated with immigration status appear to dissipate over time, as foreign-born U.S. residents’ health tends to decline with their duration of residence in the U.S. [9,10,11]. More recently, research has begun to “disentangle” these inconsistencies by examining differences in health outcomes by legal citizenship status—that is, native (i.e., born in the U.S.; born in Puerto Rico, Guam, the U.S. Virgin Islands, or Northern Marianas; and born abroad of U.S. citizen parent or parents), naturalized (i.e., foreign-born lawful permanent residents that have been granted U.S. citizenship), lawful permanent residents (i.e., non-citizens that have been granted Green Cards), temporary lawful residents (i.e., foreign nationals that have been granted visas to visit the U.S. for a limited time), and undocumented residents. Findings from a California-based study indicate that naturalized, lawful permanent residents (LPR), and undocumented residents generally report worse health outcomes than native residents. However, LPRs and undocumented residents (i.e., non-citizens), who do not have the same level of access to health insurance and other health-protective resources as naturalized and native citizens, reported the worst health outcomes. Further, these health disparities among non-citizens were largely a function of income and education inequities [12]. Additional California-based research examining the health status of naturalized citizens compared to LPRs and undocumented residents reported worse health outcomes for those that do not have citizenship status [13].

Given this evidence that non-citizens generally experience worse health outcomes than naturalized and native citizens, it would stand to reason that U.S. non-citizens (i.e., permanent legal residents, temporary legal residents, and undocumented residents) may experience disparate deleterious COVID-19-related impacts [14,15]. Despite this, nativity and citizenship status are not included in the Centers for Disease Control and Prevention (CDC) coronavirus case infection and investigation reporting forms [15]. Yet there are over 24 million non-citizens in the U.S. [9], nearly 7% of the US population [16,17]. The absence of this critical data could ultimately mask inordinate COVID-19 morbidity and mortality within non-citizen communities, and curtail critical vaccine and treatment resource delivery to these underserved communities.

### 1.1. COVID-19-Related Risk among U.S. Non-Citizens

The U.S. non-citizen population consists of lawful permanent residents (12.3 million, 49%), temporary lawful residents (2.2 million, 9%), and undocumented residents (10.5 million, 42%), with racial and ethnic minority persons representing the majority of the total immigrant population [18]. Non-citizens are generally older than the native population (median ages 38.2 and 36.1 years, respectively), though they are younger than the naturalized population (51.4 years) [17,18]. Many U.S. non-citizens are situated in a precarious nexus of socioeconomic resource inequities and associated health disparities. For instance, U.S. non-citizens are often segmented into the lowest socioeconomic strata due to lower wages and limited occupational options compared to naturalized and native U.S. citizens [14]. These socioeconomic challenges are illustrated by lower median household income ($51,401) and higher poverty rates (17.4%) among non-citizens compared to naturalized ($65,005; 9.9%) and native ($64,021; 11.4%) populations [15,17]. Such socioeconomic resource limitations are concerningly causative of some of the lowest rates of access to health insurance, healthcare, and preventive services, and thus contribute to higher rates of unmet medical needs among non-citizens in the U.S. [14,15,19]. Further, extant research has documented troubling associations between income and COVID-19 mortality [20,21]. Thus, existing socioeconomic disparities that preceded the pandemic may place non-citizens at heightened risk for COVID-19-related mortality.

In an effort to offset socioeconomic difficulties, many U.S. immigrant households have developed collaborative strategies that leverage extended family resources. For example, immigrant households often double-up and include extended family or non-relatives, thus increasing household size, to pool resources and ease financial burdens associated with high costs of living [14,22,23,24]. Importantly, the non-citizen household size on average (3.14 persons) tends to be larger than those of naturalized (2.86) and native (2.34) U.S. citizens, and greater household size is associated with heightened exposure to respiratory diseases transmitted through aerosol and respiratory droplets (e.g., tuberculosis and influenza) [15,17,25,26]. Recent research has consequently confirmed associations between COVID-19 morbidity and mortality and household size, raising additional concerns regarding non-citizens’ potentially elevated risk for COVID-19 exposure, complications, and death [27].

### 1.2. Systemic Racism and Social Determinants of COVID-19-Related Health Disparities

The root causes of these citizenship-related and broader racial and ethnic health disparities in the U.S. are ingrained in systemic racism, wherein underserved racial and ethnic minority populations are disproportionately affected by policies and practices that unevenly structure access to socioeconomic opportunities and health-promoting resources (e.g., education, healthcare) that advance health and wellbeing [28,29,30]. These systemic inequities are linked to the social determinants of health, which are the social and economic conditions—e.g., income, household size, citizenship status—associated with community health and wellbeing [31,32,33]. Inequitable access to financial resources (e.g., jobs, lending power), for example, is inextricably linked to race, ethnicity, and citizenship status. In turn, these sociodemographic factors are associated with inequitable access to health-promoting resources [14,19,32]. It is thus increasingly understood that underrepresented persons suffering from socioeconomic inequities experience poor life expectancy and a litany of health disparities [31,32,33].

### 1.3. Study Aims

To explore the impacts of COVID-19 on non-citizen residents, we examined COVID-19 mortality rates within New York City (NYC) and Los Angeles (LAC)—two major metropolitan U.S. cities with large Black, Latinx, and immigrant populations—to identify racial, ethnic, and citizenship-related disparities in these locations. We chose to examine NYC and LAC because they (1) have the two largest non-citizen populations in the U.S. (1.38 million and 717,774 non-citizens, respectively) [17]; and (2) have well-documented COVID-19-related racial and ethnic health disparities. For example, as of January 15, 2021, the COVID-19 mortality rate per 100,000 persons in New York City was 260.42 for Black persons, 280.82 for Latinx persons, and 142.16 for White persons [34]. On this same date in Los Angeles, the mortality rate per 100,000 persons was 119 for Black persons, 192 for Latinx persons, and 69 for White persons [35].

Yet, despite these important similarities, NYC and LAC also possess notable differences in key domains including geographic distribution, percent Black and Latinx (e.g., NYC has a higher proportion of Black residents while LAC has a higher proportion of Latinx residents) that would make running our analyses in both contexts an apt opportunity to test the generalizability of potential disparities in COVID-19 impacts on U.S. non-citizen residents. In other words, if we found similar patterns in NYC and LAC, that would provide compelling evidence that non-citizenship is an important factor in U.S. COVID-19 mortality. We hypothesized that, while controlling for established social determinants of COVID-19 mortality (i.e., race and ethnicity, sex, age, income, household size) [36,37], areas with greater proportions of non-citizen residents would experience higher COVID-19 mortality rates compared to areas with greater proportions of native and naturalized citizens in both the NYC and LAC contexts.

## 2. Methods

### 2.1. Data

**Figure 1:**
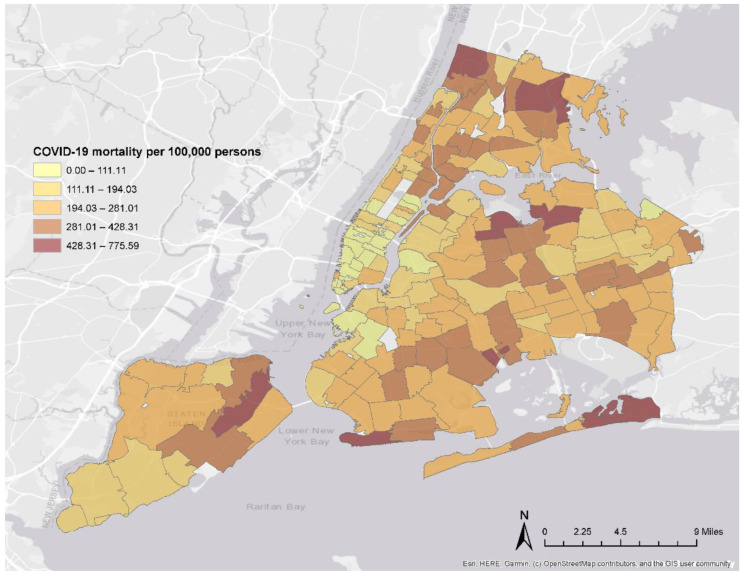
New York City ZIP Code Tabulation Area COVID-19 Mortality Rates.

**Figure 2:**
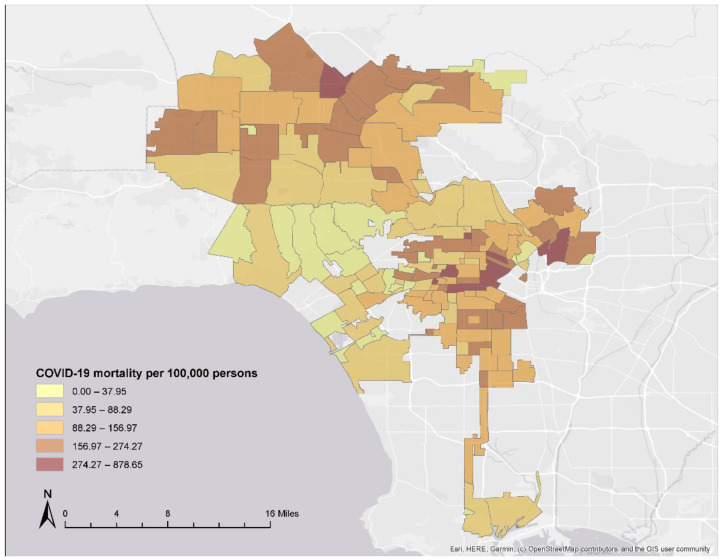
Los Angeles City/Community COVID-19 Mortality Rates.

We collected NYC Department of Health and Mental Hygiene COVID-19 mortality rates per 100,000 persons of the population for all 177 NYC ZIP Code Tabulation Areas (ZCTA) reporting COVID-19 data from 29 February 2020 to 15 January 2021 (Figure 1). NYC ZCTAs ranged from 0.05 to 13.68 square miles. For LAC, Los Angeles County Department of Public Health (LADPH) COVID-19 mortality rates per 100,000 persons were collected for all 134 LAC Cities/Communities with available COVID-19 data from 1 March 2020 to 15 January 2021 (Figure 2). Cities/Communities are Los Angeles County Census-designated places used by LADPH to report health indicators and outcomes. There were between 5 and 63 census tracts within LAC City/Community boundaries, each of which ranged from 0.08 to 15.05 square miles. To calculate LAC City/Community-level census variables, we mapped all census tracts within each City/Community boundary. All census tracts nested perfectly within City/Community boundaries, thus allowing us to accurately determine population estimates without applying complex data reapportionment methods. For LAC, census tract-level data were summed to calculate population counts in each City/Community then population density was calculated per square mile and percent population was calculated for race and ethnicity, sex, age, and citizenship variables. Income and household-level variables were calculated by summing census data and computing the mean for each City/Community.

Based on known social determinants of COVID-19 mortality [36,37], we collected 2018 American Community Survey 5-Year Estimates for ZCTA (NYC) and census tract-level (LAC) data including percent Black, percent Latinx, population density, percent female, percent over 55 years of age, median household income, and the average number of people per household. We also collected percent naturalized and non-citizen residents in NYC ZCTAs and LAC census tracts aggregated to Cities/Communities to explore associations between citizenship on COVID-19 mortality [17]. We then summed percent Black and percent Latinx census data at the ZCTA (NYC) and census tract (LAC) levels into a single Black and Latinx variable (i.e., percent Black + percent Latinx) to account for (1) the considerable overlap of Black and Latinx residents in NYC and LAC census boundaries, (2) the differing racial compositions in these geographies, and (3) small populations in some census boundaries that preclude separate examinations of racial and ethnic groups within the NYC and LAC geographies [29]. All COVID-19 mortality and census variables were mapped in ArcMap 10.5. This study utilized publicly available data and was determined to be exempt from ethical compliance by the Chapman University Institutional Review Board.

### 2.2. Statistical Analyses

We calculated descriptive statistics at the ZCTA (for NYC) and City/Community (for LAC) levels. To examine the effects of citizenship vs. non-citizenship in our cross-sectional study, we developed two linear multiple regression models (NYC and LAC) to examine associations between the percent of non-citizens and COVID-19 mortality per 100,000 persons. Informed by the available literature [36,37], our theory-driven model entered data in 3 blocks to control for known social determinants of COVID-19 mortality while isolating citizenship in the final block. Block 1 controlled for percent Black and Latinx; Block 2 controlled for population density, percent female, percent over 55 years of age, the average number of people per household, median household income, and percent naturalized citizens; and percent non-citizens was entered in Block 3 to test associations between percent non-citizens and COVID-19 mortality. Native U.S. citizens (i.e., census respondents that selected “Yes, born in the United States”; “Yes, born in Puerto Rico, Guam, the U.S. Virgin Islands, or Northern Marianas”; and “Yes, born abroad of U.S. citizen parent or parents”) served as our reference variable. Variance inflation factors below 2.5 in the NYC model and 5.9 in the LAC model revealed that multicollinearity was not a concern. All analyses were conducted in SPSS v25 (IBM Corp. Released 2017. IBM SPSS Statistics for Mac, Version 25.0. IBM Corp.: Armonk, NY, USA).

## 3. Results

Table 1 provides NYC ZCTA-level and LAC City/Community-level descriptive statistics. While the geographic and population distributions of NYC and LAC are quite different, results indicate these cities are markedly similar at the community-level in terms of population sociodemographic composition, including sex, age, household size, median household income, and naturalized and non-citizen residents. However, NYC has a larger Black population and LAC has a larger Latinx population, with strikingly different population density and cumulative COVID-19 deaths per 100,000 persons in these two cities. These similarities and differences made these two distinct cities ideal for testing our COVID-19 mortality and citizenship status hypothesis, as population density, race and ethnicity, sex, age, household size, and income are known to associate with COVID-19 mortality [36,38].

To test our non-citizenship-mortality hypothesis, we conducted multiple regression analyses with COVID-19 mortality as the dependent variable, separately for NYC and LAC, to examine the independent effects of percent non-citizen residents while controlling for other demographic factors. Results indicated that our models accounted for 47% and 24% of the variance in COVID-19 deaths per 100,000 persons, in NYC and LAC, respectively.

For the NYC model (Table 2), ZCTA-level increases in percent Black and Latinx, percent over 55 years of age, and percent non-citizens significantly associated with COVID-19 death rate increases (*p* < 0.05). Increasing population density and median household income significantly associated with COVID-19 death rate decreases (*p* < 0.05).

In the LAC model (Table 2), City/Community-level increases in percent over 55 years of age and percent non-citizens significantly associated with COVID-19 death rate increases (*p* < 0.05). Notably, LAC City/Community increases in percentage Black and Latinx significantly associated with COVID-19 death rate increases in Block 1 of our model, but did not in Blocks 2 and 3 after accounting for additional social determinants of health variables and percent non-citizen residents.

## 4. Discussion

While controlling for known covariates of COVID-19 mortality [1,36,37], our results suggest that as postulated, having a greater presence of non-citizen residents was significantly associated with higher rates of COVID-19 mortality in both NYC and LAC. Further, while existing research and public health surveillance have revealed that Black and Latinx U.S. residents are at elevated risk for COVID-19-related racial and ethnic health disparities during this pandemic [1], our findings suggest that communities with large shares of non-citizens may carry a higher level of COVID-19 mortality risk. Thus, this is the first population-level study to our knowledge to explore non-citizen status as a critical but potentially underrecognized risk factor for COVID-19 mortality. Additionally, while NYC and LAC made notable efforts to increase viral testing and hospital resources to reduce the disparate racial and ethnic impacts of the COVID-19 pandemic [29], our data suggest that within these large, ethnically and culturally diverse cities, communities with greater numbers of non-citizen residents continued to experience striking disparities in COVID-19 mortality—signifying a possible need to prioritize delivery of essential healthcare resources (e.g., COVID-19 testing and vaccines, healthcare services) and broader public benefits (e.g., financial resources) to non-citizen residents during both ongoing and future pandemics.

These disproportionately high COVID-19 mortality rates affecting NYC and LAC communities with heavier proportions of non-citizen residents may be a function of the socioeconomic resource limitations often experienced by U.S. non-citizens. For instance, lower income areas have been shown to have higher COVID-19 mortality rates [39]. Further, nearly 6 million non-citizens were not eligible for Coronavirus Aid, Relief, and Economic Security (CARES) Act benefits [40]. Therefore, it may be unsurprising that non-citizen resident communities appear to endure higher COVID-19 mortality as median earnings are generally lower among non-citizen residents versus native and naturalized U.S. residents [14,15,17,27]. Indeed, in our analyses, median household income appeared to be associated with higher COVID-19 mortality in NYC, with the addition of the percent non-citizen resident variable further strengthening our models. However, median household income was not significant in the LAC context, potentially signifying that legal citizenship status may be a critical indicator of COVID-19 mortality risk.

The process of doubling-up, wherein non-citizen households pool financial resources, frequently leading to a larger household size compared to naturalized and native resident households, may have presented additional challenges, as household size is a known determinant of respiratory disease transmission [15,25,26]. Thus, household size may have further increased non-citizens’ chances of COVID-19 exposure and subsequent infection and mortality. For example, the available research indicates that larger household size among Black and Latinx households may be a potential mechanism of COVID-19 exposure [41], and extant data indicates the majority of non-citizens are underrepresented racial and ethnic minorities [18]. While household size was not statistically significant in our models at the ecological level, larger household size may have affected non-citizen residents’ ability to physically distance themselves from other household members [14,22,23,24].

Employment status may be another indicator of increased COVID-19 mortality risk. Foreign-born U.S. residents comprise 18% of essential retail and wholesale, 26% of manufacturing, 17% of transportation, 27% of agricultural, and 13% of postal jobs. In aggregate, 69% of foreign-born and 74% of undocumented U.S. residents are essential workers [42]. During the H1N1 pandemic in the U.S., “Spanish language-dominant” Latinx U.S. residents were generally more susceptible to viral exposure, partly as a result of inequitable access to employment that afforded work from home opportunities, with about 81% of Spanish speaking participants in a national study indicating that they would have “difficulty staying home from work for 7–10 days” and 58% indicating they “could lose their job or business if not able to go to work” [6]. With the lack of COVID-19-related relief programs, such as CARES, for many non-citizens [40], it may have been difficult to stay at home to avoid disease transmission associated with essential worker environments. Further, in the New York and Los Angeles contexts, foreign-born residents comprise 33% and 32% of the healthcare workforce, respectively. Thus, foreign-born essential workers “are disproportionately represented in the labor force” [42].

The lack of a universal healthcare system in the U.S., which places underrepresented U.S. residents with limited socioeconomic opportunities at a significant healthcare disadvantage, may have encumbered non-citizens’ healthcare resource access. For instance, underrepresented U.S. residents (e.g., Black, Latinx, and non-citizen residents) are less likely to have a usual source of care and have more unmet medical needs than residents in affluent White communities [14,15,19,43]. In the context of the COVID-19 pandemic, emerging research has similarly highlighted the presence of these inequities. For example, low-income Black and Latinx communities were challenged by inequitable geographic access to (1) licensed and ICU hospital beds during the April 2020 pandemic peak in New York City [29], and (2) COVID-19 viral testing resources in Los Angeles City [44]. Thus, due to inequities in health-protective resources for many racial and ethnic minority residents in the context of cities such as New York and Los Angeles, residents lacking citizenship status may be at greater risk of COVID-19 infection and death due to restricted resource access stemming from their non-citizen status relative to residents of other NYC and LAC communities.

State and local policies may affect non-citizens’ access to public benefits (e.g., CARES, healthcare resources). For example, U.S. non-citizens of color are unequally targeted for deportation—95% of non-citizens deported are from Latin American countries—which may raise hesitancy when accessing public benefits [45,46]. As De Trinidad Young and Wallace [45] argue, the dual processes of criminalization and integration at various levels exacerbate health and other disparities for non-citizens. Many U.S. cities that provide protections against deportation and prosecution, such as NYC and LAC, have also adopted policies that improve undocumented residents’ access to public benefits that are vital to reducing pandemic-related disparities. However, the protections in services afforded to non-citizens by sanctuary cities may be limited by state-level policies. For example, New York City has implemented several policies that produce a more inclusive environment that provides non-citizens access to health and social service benefits, education, and employment [23]. In addition, Local Law 228 prohibits the use of City resources and staff for immigration enforcement. However, New York State laws have implemented a series of criminalizing policies that may discourage non-citizens from accessing public benefits, including law enforcement collaboration with federal immigration enforcement. In contrast, Los Angeles County provides a range of inclusive health, education, and employment services while affording a safe environment for non-citizens that is reinforced by California State policies against cooperating with federal immigration services [45]. Therefore, scholars have characterized New York State as having a “deportable inclusion” policy context, and California as a “proactive inclusion” context [45].

The federal context may also encumber non-citizen residents’ access to lifesaving financial support and healthcare resources. For example, non-citizen access to public benefits (e.g., Medicaid, Supplemental Nutrition Assistance Program (SNAP), housing assistance) is limited by the five-year bar—a five-year waiting period required for non-citizens to meet public benefit eligibility requirements—despite non-citizens being more vulnerable to healthcare and housing inequities related to socioeconomic difficulties [15]. The 2020 public charge amendments stating that immigrants receiving federally-funded cash and non-cash benefits, including Medicaid, SNAP, and housing assistance would be considered ineligible for permanent residence may have also created confusion and apprehension among non-citizens about what services they could legally and safely access [15]. A report from the Center for Migration Studies of New York provides evidence that Trump-era amendments to the public charge rule “perpetuated fear among immigrant communities” in the context of accessing public benefits and services, including healthcare services [47]. Further, the proposed change to the 2020 U.S. Census to include a citizenship question reflects an enduring anti-immigration sociopolitical climate that may have impacted non-citizens’ perceptions of safety around accessing services during the pandemic.

Thus, in the deportable inclusion context of New York, non-citizens may have been deterred from accessing critical public benefits, such as healthcare services, due to the perceived “threat of surveillance, policing, and deportation” [45]. Furthermore, in both NYC and LAC contexts, federal laws—e.g., the 5-year bar and public charge rule—that unequally structure access to public benefits for non-citizens may have also deterred non-citizens from accessing critical care during the study period. Based on these potential underlying factors (e.g., low income, inequitable healthcare access), we contend from our data that U.S. non-citizen residents may reside in communities that bear systemic inequities in the social and built environment that suppress health and increase the risk for COVID-19 mortality. Yet, given the limited research examining the relationships between community-level social determinants and health outcomes among U.S. non-citizen residents, further research is now required to determine whether non-citizen residents experience poorer health and well-being as a function of their environments.

Overall, our findings, in agreement with our social determinants of the health-informed model and reflecting the challenges of systemic racism, indicate that U.S. residents living in communities with large shares of non-citizens may be at greater risk for COVID-19-related health disparities; potentially due to key social factors such as inequitable access to financial and health-promoting resources [14,24]. Therefore, targeting critical healthcare resource delivery and health messaging toward curtailing the disparate impacts of COVID-19, as well as a litany of health disparities (e.g., tuberculosis, influenza), in communities with large shares of non-citizen residents plagued by socioeconomic resource limitations may serve to reduce these health disparities.

There are several limitations to the current study. First, while data concerning the share of non-citizen residents who were permanent legal residents, temporary legal residents, and undocumented residents in NYC and LAC were not available, this data could have further enhanced our understanding of how non-citizen status is associated with COVID-19 mortality rates in our target cities. Second, mortality rates disaggregated by race and ethnicity may have enhanced our ability to uncover COVID-19 mortality disparities in NYC and LAC. Third, controlling for the prevalence of ZCTA (NYC) and City/Community-level (LAC) comorbidities (e.g., hypertension, obesity) known to exacerbate COVID-19 severity may have provided a more thorough understanding of COVID-19 mortality rate associations with percent non-citizens. Fourth, our study did not account for the differential timing and intensity of COVID-19 in NYC and LAC. Fifth, future studies may benefit from spatial analyses examining non-citizenship in the context of healthcare resource access (e.g., hospital and primary care resources), employment status, and other community-level mechanisms. Sixth, it is important to note that our results represented ecological-level associations and not individual disparities by citizenship status. Finally, we were unable to make causal inferences between our study variables due to our cross-sectional study design.

## 5. Conclusions

The myriad socioeconomic challenges that non-citizens are faced with in U.S. urban settings, dovetailed by federal policies that threatened prospects of permanent residency, may have ultimately discouraged help-seeking behaviors among non-citizens. In our findings, the percent of non-citizens appeared to associate with increased COVID-19 mortality rates in both NYC and LAC, irrespective of state and local policy contexts, indicating federal policies and other individual- (e.g., legal, employment status) and community-level (e.g., geographic access to COVID-19 treatment resources) factors may have encumbered non-citizens’ access to critical public benefits, such as financial and healthcare resources [29,47]. Although many U.S. cities have implemented policies and practices that seek to expand non-citizen access to public benefits, additional policies that (1) develop public health surveillance for non-citizen residents, (2) improve public health messaging and overall healthcare resource access for non-citizens [15], and (3) address inequitable policies and practices rooted in systemic racism that have contributed to glaring health disparities may help to curtail disparate COVID-19 mortality rates and advance health and wellbeing for the many U.S. residents that do not experience the benefits of citizenship.

## Figures and Tables

**Table 1:** NYC ZCTA-level and LAC Community-Level Descriptive Statistics.

	NYC		LAC	
	Mean	95% CI	Mean	95% CI
COVID-19 mortality per 100 K	233.95	216.26, 251.63	124.49	106.61, 142.36
Percent Black & Latinx	45.82	41.29, 50.36	52.81	47.73, 57.90
Population density per square mile	45,174.65	40,462.63, 49,886.68	13,391.15	11,822.11, 14,960.20
Percent female	52.16	51.78, 52.54	50.95	50.54, 51.35
Percent over 55 years of age	24.27	23.37, 25.17	24.94	23.82, 26.06
Average household size	2.64	2.56, 2.71	2.82	2.72, 2.92
Median household income	73,674.02	68,254.57, 79,034.47	71,896.58	65,996.95, 77,796.22
Percent naturalized residents	19.95	18.70, 21.21	17.37	16.39, 18.34
Percent non-citizen residents	15.35	14.30, 16.41	17.55	15.97, 19.14

**Table 2:** Regression of predictor variables on NYC and LAC COVID-19 mortality rates.

	NYC				LAC			
	*R* ^2^	β	*B*	(95% CI)	*R* ^2^	β	*B*	(95% CI)
**Block 1**	0.208 ***				0.092 ***			
Percent Black & Latinx		0.390 ***	1.522	(0.851, 2.193)		−0.068	−0.240	(−1.513, 1.033)
**Block 2**	0.460 ***				0.213 ***			
Population density		−0.153 *	−0.001	(−0.001, −0.000)		0.089	0.001	(−0.002, 0.004)
Percent female		0.078	3.609	(−2.566, 9.784)		0.064	2.810	(−4.279, 9.899)
Percent over 55 years of age		0.352 ***	6.901	(3.892, 9.910)		0.388 **	6.198	(2.041, 10.355)
Household size		−0.019	−4.488	(−43.421, 34.445)		0.155	27.580	(−17.255, 72.415)
Median household income		−0.196 *	−0.001	(−0.001, −0.000)		−0.294	−0.001	(−0.002, 0.000)
Percent naturalized citizens		0.096	1.354	(−0.907, 3.615)		0.032	0.592	(−3.163, 4.347)
**Block 3**	0.472 *				0.237 *			
Percent non-citizens		0.164 *	2.745	(0.309, 5.181)		0.409 *	4.609	(0.498, 8.720)

Boldface indicates statistical significance (* *p* < 0.05 ** *p* < 0.01 *** *p* < 0.001). Adjusted *R*^2^ and final model β (standardized coefficients) and *B* (unstandardized coefficients) reported. Confidence intervals were calculated for *B* (unstandardized coefficients).

## Data Availability

Publicly available datasets were analyzed in this study. This data can be found here: https://www1.nyc.gov/site/doh/covid/covid-19-data-totals.page (accessed on 16 January 2021); http://publichealth.lacounty.gov/media/coronavirus/data/index.htm#graph-deathrate (accessed on 16 January 2021).
